# Genome-Wide Characterization of the *ABI3* Gene Family in Cotton

**DOI:** 10.3390/genes16080854

**Published:** 2025-07-23

**Authors:** Guoyong Fu, Yanlong Yang, Tahir Mahmood, Xinxin Liu, Zongming Xie, Zengqiang Zhao, Yongmei Dong, Yousheng Tian, Jehanzeb Farooq, Iram Sharif, Youzhong Li

**Affiliations:** 1 Xinjiang Cotton Technology Innovation Center, Xinjiang Key Laboratory of Cotton Genetic Improvement and Intelligent Production, National Cotton Engineering Technology Research Center, Cotton Research Institute of Xinjiang Uyghur Autonomous Region Academy of Agricultural Sciences, Urumqi 830091, China; fgyong1212@163.com (G.F.); yangyl0629@163.com (Y.Y.); 2 State Key Laboratory of Cotton Biology, Institute of Cotton Research of the Chinese Academy of Agricultural Sciences, Anyang 455000, China; tahirmtaha@hotmail.com; 3 College of Life Sciences, Xinjiang Normal University, Urumqi 830054, China; xsdyjstg@sina.com; 4 Cotton Research Institute, Xinjiang Academy of Agricultural and Reclamation Science, Key Laboratory of Cotton Biology and Genetic Breeding in the Northwest Inland Cotton Production Region, Ministry of Agriculture and Rural Affairs, Shihezi 832000, China; xiezmchy@163.com (Z.X.); tlx4109@163.com (Z.Z.); ymd_sh@sohu.com (Y.D.); tianyoushengzi@163.com (Y.T.); 5 Cotton Research Station, Ayub Agricultural Research Institute, Faisalabad 38000, Pakistan; iramsharif695@yahoo.com

**Keywords:** ABI3, *Gossypium*, gene family evolution, synteny, cis-regulatory elements, abiotic stress

## Abstract

**Background:** The B3-domain transcription factor ABI3 (ABSCISIC ACID INSENSITIVE 3) is a critical regulator of seed maturation, stress adaptation, and hormonal signaling in plants. However, its evolutionary dynamics and functional roles in cotton (*Gossypium* spp.) remain poorly characterized. **Methods**: We conducted a comprehensive genome-wide investigation of the ABI3 gene family across 26 plant species, with a focus on 8 *Gossypium* species. Analyses included phylogenetics, chromosomal localization, synteny assessment, gene duplication patterns, protein domain characterization, promoter cis-regulatory element identification, and tissue-specific/spatiotemporal expression profiling under different organizations of *Gossypium hirsutum*. **Results**: Phylogenetic and chromosomal analyses revealed conserved ABI3 evolutionary patterns between monocots and dicots, alongside lineage-specific expansion events within *Gossypium* spp. Syntenic relationships and duplication analysis in *G. hirsutum* (upland cotton) indicated retention of ancestral synteny blocks and functional diversification driven predominantly by segmental duplication. Structural characterization confirmed the presence of conserved B3 domains in all *G. hirsutum* ABI3 homologs. Promoter analysis identified key stress-responsive cis-elements, including ABA-responsive (ABRE), drought-responsive (MYB), and low-temperature-responsive (LTRE) motifs, suggesting a role in abiotic stress regulation. Expression profiling demonstrated significant tissue-specific transcriptional activity across roots, stems, leaves, and fiber developmental stages. **Conclusions**: This study addresses a significant knowledge gap by elucidating the evolution, structure, and stress-responsive expression profiles of the ABI3 gene family in cotton. It establishes a foundational framework for future functional validation and targeted genetic engineering strategies aimed at developing stress-resilient cotton cultivars with enhanced fiber quality.

## 1. Introduction

ABSCISIC ACID INSENSITIVE 3 (ABI3) is a B3 domain-containing transcription factor that acts as a central regulator in abscisic acid (ABA) signaling. The ABI3 protein is inherently unstable *in planta*, and its interaction with the E3 ubiquitin ligase AP2 facilitates ubiquitin-mediated degradation. Consequently, AP2 functions as a negative regulator of ABA signaling [1]. The ABI3 protein binds specifically to the highly conserved RY motif (CATGCA[TG]), present in numerous seed-specific promoters. This interaction, mediated by the B3 domain, is essential for ABI3 function. ABI3 plays a critical role in seed maturation, acting as a key regulator of the transition between embryo maturation and early seedling development [2,3]. Research indicates that during embryogenesis under cold-inductive conditions, ABI3 binds to the cold-memory element within the flowering gene *FLC* (FLOWERING LOCUS C) and recruits scaffolding proteins associated with active chromatin modifications. This complex facilitates the resetting of *FLC* silencing. Furthermore, in response to ABA accumulation during embryo maturation, ABI3, in conjunction with ABI5, activates *FLC* expression by binding to ABA-responsive elements within the *FLC* promoter. This dual activation of *FLC* by ABI3 ensures the vernalization requirement in each generation of Arabidopsis thaliana [2]. Additionally, ETHYLENE INSENSITIVE 2 (EIN2), a core component of the ethylene signaling pathway in Arabidopsis, fine-tunes ABA responses during seed germination and early seedling establishment. EIN2 achieves this by interacting with HOOKLESS 1 (HLS1), a putative histone acetyltransferase, thereby suppressing histone H3 acetylation on the chromatin of ABI3 and ABI5 [4].In seed maturation, ABI3 orchestrates desiccation tolerance and longevity by activating late embryogenesis abundant (LEA) proteins and small heat shock proteins (HSPs), while repressing chlorophyll retention through interactions with the stay-green gene SGR1 to ensure proper seed degreening [3,5]. Notably, ABI3 directly binds ABA-responsive cis-elements (e.g., RY motifs) to activate tonoplast aquaporins (TIP3s) and chromatin remodelers like CHR5, establishing active chromatin states for seed-specific gene expression [5,6]. During germination, ABI3 acts as a molecular sensor of ABA levels, modulating auxin signaling by repressing SHY2 to enhance primary root growth under mild dehydration, while high ABA shifts dominance to ABI5, arresting root meristem activity [7].

ABI3 is pivotal in plant responses to abiotic stresses, integrating ABA signaling with stress tolerance mechanisms. Under drought, ABI3 enhances stomatal closure and antioxidant enzyme activity by upregulating CBF4 and repressing aquaporin plasma membrane intrinsic protein 2;8 (PIP2;8) through Plant A/T-rich protein and zinc-binding protein 4 (PLATZ4), thereby improving water retention [8]. In moss (*Physcomitrium patens*), ABI3-SnRK2 cascades elevate non-photochemical quenching (NPQ) via LHCSR induction and xanthophyll cycle modulation, highlighting its conserved role in photoprotection [9]. Transgenic overexpression of ScABI3 from the desiccation-tolerant moss *Syntrichia caninervis* in alfalfa enhances salt tolerance by activating stress-responsive genes (e.g., PYL/PYR, SnRK2s) and improving photosynthetic efficiency [10]. These findings underscore ABI3’s versatility in stress adaptation, spanning chromatin remodeling and transcriptional networks [11,12]. Harnessing ABI3 orthologs or editing its regulatory elements (e.g., *TaABI3-A1Hap1* in wheat) offers promising strategies for engineering crops with enhanced resilience to climate extremes, bridging evolutionary insights from bryophytes to angiosperms [9,11,12].

Cotton is an important cash crop, and cotton fiber is one of the textile industry’s most important natural raw materials. Land-based cotton accounts for more than 95% of the world’s cotton fiber production and is the most widely grown cotton species worldwide [13]. Cotton fibers originate from a type of unicellular trichome in the epidermal cells of the ovule, making it an ideal model for studying cell elongation and differentiation [14]. Fiber development is regulated by a variety of hormonal effects, with hormones such as gibberellins, brassinosteroids, and auxin thought to promote fiber development, while ABA and cytokinins are known to inhibit fiber development [15]. GbEXPA2 expression was significantly upregulated during cotton fiber elongation. Promoter fragments of varying lengths from GbEXPA2 were fused to the GUS reporter gene and introduced into Arabidopsis thaliana. Following exogenous ABA application, GUS activity exhibited a significant downregulation. These findings suggest that ABA may inhibit fiber elongation [16]. ABI3 is a central component of the ABA signaling pathway. The genomic distribution of ABI3 in cotton and its prospective role in fiber development remain unexplored.

This study systematically investigated the evolutionary dynamics and functional diversification of the ABI3 gene family across 26 plant species, focusing on phylogenetic relationships and quantitative divergence. Comparative genomic analyses were conducted to delineate chromosomal localization patterns of ABI3 homologs among eight cotton species (*Gossypium* spp.), revealing lineage-specific distribution trends. Syntenic relationships and gene duplication events within the ABI3 family were rigorously examined between *G. hirsutum* (upland cotton) and seven additional cotton species, highlighting conserved and divergent evolutionary trajectories. Furthermore, structural characterization of the ABI3 family in *G. hirsutum* encompassed an in-depth analysis of gene architecture, conserved functional domains, and cis-regulatory elements within promoter regions, identifying critical motifs associated with stress responsiveness and hormonal regulation. Finally, tissue-specific expression profiling of ABI3 genes in *G. hirsutum* was performed to elucidate differential transcriptional activity across distinct developmental stages and organs. This comprehensive approach provides novel insights into the evolutionary conservation and functional specialization of ABI3 in cotton species.

## 2. Materials and Methods

### 2.1. Systematic Identification and Characterization of the ABI3s Gene Family

The eight cotton genome files, *Gossypium herbaceum* (WHU, version 1.0, Ghe), *Gossypium arboreum* (CRI, version 1.0, Ga), *Gossypium raimondii* (JGI, version 2.0, Gr), *G. hirsutum* (CRI, version 1.1, Gh), *Gossypium barbadense* (HAU, version 1.1, Gb), *Gossypium tomentosum* (HGS, version 1.0, Gt), *Gossypium mustelinum* (JGI, version 1.0, Gm), and *Gossypium darwinii* (HGS, version 1.0, Gd), were downloaded from CottonMD (https://yanglab.hzau.edu.cn/CottonMD, accessed on 8 February 2024) [17] (Supplementary Table S1). Genomic data for *Marchantia polymorpha*, *Physcomitrella patens*, *Fontinalis antipyretica*, *Entodon seductrix*, *Hypnum curvifolium*, *Anthoceros angustus*, *Anthoceros agrestis*, *Anthoceros punctatus*, *Chlorokybus atmophyticus*, *Chara braunii*, *Mesotaenium endlicherianum*, *Spirogloea muscicola*, *Selaginella moellendorffii*, *Picea abies*, *Oryza sativa*, *Amborella trichopoda*, *Arabidopsis thaliana*, and *Aristolochia fimbriata* were obtained from different data databases (Supplementary Table S1). The Hidden Markov model (HMM; version 3.0) profile for the ABI3 domain (Pfam accession: PF02362) was retrieved from the Pfam database (Pfam: Home page (xfam.org, accessed on 8 February 2024)) [18]. Using HMMER 3.0 (http://www.hmmer.org/, accessed on 8 February 2024) with default parameters [19], we identified putative ABI3 family genes across 26 species. Redundant sequences (E-value > 1 × 10^−50^) were filtered out. Candidate genes were further validated against the Conserved Domain Database (CDD) at NCBI to confirm ABI3 domain integrity. After this screening pipeline, 1927 high-confidence ABI3 genes were retained for subsequent analysis. The distribution of ABI3 genes among the 26 species was then quantified.

### 2.2. Multiple Sequence Alignment Coupled with Molecular Phylogeny Inference

The protein sequences of 1927 ABI3 homologs from 26 species were aligned using MAFFT (v7.520) and subsequently trimmed with TrimAl (v1.4.rev15) to remove poorly aligned regions [20,21]. A maximum-likelihood phylogenetic tree was then constructed with IQ-TREE (v2.1.2) under the best-fit model selected by ModelFinder, followed by visualization and annotation using iTOL (v6.7) [22,23,24,25]. We used the JTT+F+R7 model of IQ-TREE software (v2.1.2) to construct the phylogenetic tree with maximum likelihood by ultrafast bootstrap (1000 replicates). The same method was used to build a phylogenetic tree among Ghe, Ga, Gr, Gh, Gb, Gt, Gm, and Gb in the phylogenetic tree.

### 2.3. Chromosomal Localization

Chromosomal localization patterns were conducted via TBtools (v2.089) software [26]. “Gene Location Visualize from GTF/GFF” module, Gene density was prepared using the “Gene Density Profile” module.

### 2.4. Gene Family Collinearity Analysis

Collinearity analysis of the ABI3 gene family was conducted between *G. hirsutum* (Gh) and seven diploid/allotetraploid cotton species (*G. arboreum* (Ga), *G. raimondii* (Gr), *G. barbadense* (Gb), *G. thurberi* (Gt), *G. mustelinum* (Gm), *G. davidsonii* (Gd), *G. herbaceum* (Ghe)) using the “One Step MCScanX” module in TBtools with default parameters. Subsequent synteny visualization was systematically performed through the integrated workflow: (1) Data preprocessing via “File Merge for MCScanX” to compile genome-wide gene positions; (2) Format standardization using “File Transformation for MicroSynteny Viewer” with GenePair; and (3) Sequence statistics generation through “Fasta Stats” to validate input integrity. The final Advanced Circos were generated with adaptive scaling to illustrate macro-syntenic relationships between Gh and each comparator genome.

### 2.5. Gene Structure, Conserved Structural Domains, and Phylogenetic Tree

Protein sequences of Gh were retrieved using the “GXF Sequences Extract”, “Batch Translate CDS to Protein”, and “Fasta Extract” modules in TBtools. Conserved motifs were identified via the MEME Suite (https://meme-suite.org, accessed on 8 February 2024) with parameters set to detect 10 motifs (default: E-value < 1 × 10^−5^, width range 6–50 residues).

Gene structures were annotated by parsing genome annotation files (GFF3) using the “Visualize Gene Structure” module. Conserved domains were predicted via NCBI’s Conserved Domain Database (CDD) with an E-value threshold of 1 × 10^−3^ [27].

Evolutionary relationships, domain architectures, and motif distributions were integrated using the “Gene Structure View (Advanced)” module. The preconstructed maximum likelihood phylogenetic tree, domain annotations, and motif patterns were aligned and visually optimized for clarity.

### 2.6. Gh_ABI3s Cis-Regulatory Element Analysis and Visualization

Promoter sequences (2000 bp upstream of transcription start sites) were submitted to the PlantCARE database (https://bioinformatics.psb.ugent.be/webtools/plantcare/html/, accessed on 8 February 2024) using the “Search for CARE” tool for in silico identification of cis-regulatory elements [28]. Raw predictions were curated by removing ubiquitous core promoter elements (e.g., TATA-box, CAAT-box) through regular expression filtering (TATA\W+, CAAT\W+). Functional classification of retained elements was performed according to the hierarchical framework established by Mengarelli et al. (2021) [29], categorizing motifs into (1) phytohormone responsive (e.g., ABRE, AuxRR-core), (2) abiotic and biotic stresses (e.g., MBS, DRE core, ARE), and (3) plant growth and development (e.g., G-box, AE-box).

A customized R script (R PromoterAnalysis.txt) adapted from Mengarelli et al.’s workflow was implemented for motif distribution visualization. Color gradients were assigned based on functional categories, and non-informative elements were excluded to enhance interpretability.

### 2.7. Expression Analysis of Gh_ABI3 in Different Tissues

J02-508 and Zhong RI-015 are two land cotton materials that exhibit significant differences in fiber quality [30,31]. To investigate the expression levels of the ABI3 gene family during the development of primary tissues and fibers in cotton, we analyzed the expression levels of the ABI3 gene family in both materials across different tissues and developmental stages of fiber. All raw RNA-sequencing data were submitted to the NCBI SRA database under the project accession number PRJNA634606.

## 3. Results

### 3.1. Comparative Phylogenomics of the ABI3 Gene Family: Identification and Evolutionary Analysis

**Figure 1:**
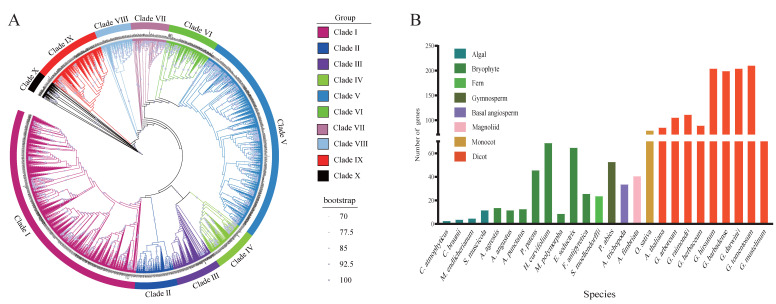
ABI3s are highly conserved across various species. (**A**) A phylogenetic tree was constructed for 1927 ABSCISIC ACID INSENSITIVE3 proteins from 26 species. (**B**) The number of SCPLs in 26 different species.

To elucidate the evolutionary trajectory and functional diversification of the ABI3 family of proteins in plants, we systematically analyzed 26 representative species spanning major plant lineages, including Algal species (*Chlorokybus atmophyticus*, *Chara braunii*, *Mesotaenium endlicherianum*, *Spirogloea muscicola*), Bryophytes (*Anthoceros agrestis*, *Anthoceros angustus*, *Anthoceros punctatus*, *Physcomitrella patens*, *Hypnum curvifolium*, *Marchantia polymorpha*, *Entodon seductrix*, *Fontinalis antipyretica*), Ferns (*Selaginella moellendorffii*), Gymnosperms (*Picea abies*), Basal angiosperms (*Amborella trichopoda*), Magnoliids (*Aristolochia fimbriata*), Monocots (*Oryza sativa*), and Eudicots (*Arabidopsis thaliana*; *Gossypium* spp.: *G. hirsutum*, *G. barbadense*, *G. arboreum*, *G. herbaceum*, *G. tomentosum*, *G. darwinii*, *G. raimondii*, *G. mustelinum*). Through Hidden Markov Model (HMM) profiling and conserved domain validation (CDD), we identified 1927 ABI3 homologs for subsequent phylogenetic analyses. Maximum-likelihood phylogenetic reconstruction using IQ-TREE resolved the ABI3 family into 10 distinct clades (Figure 1A, Supplementary Table S2). Clade I (583 genes) and Clade V (515 genes) emerged as the most gene-rich clades, collectively representing 57.0% of total ABI3 members. In contrast, Clade X (49 genes) and Clade VIII (87 genes) exhibited a significant reduction in the number of genes, with Clade I containing 11.89-fold more genes than Clade X. Clade I contained the highest number of species (26 species). Within Clade I, *Gossypium* species accounted for 74.27% of the total. Species Gb, Gt, Gm, and Gd each represented an equal proportion of 12% (70/583). *Mesotaenium endlicherianum* exhibited the lowest proportion at 0.17% (1/583), while *Arabidopsis thaliana* accounted for 3.95%. In Clade X, 12 species possessed the ABI3 gene. Within this clade, the *Arabidopsis thaliana* gene showed the highest proportion at 8.16%. *Gossypium* species collectively accounted for 68.53%, with Gh/Gb/Gt/Gm/Gd exhibiting the highest individual proportion among cottons at 12.24% (6/49). *Aristolochia fimbriata* and *Amborella trichopoda* showed the lowest proportion, each at 2.04% (1/49). Clade VII displayed the lowest representation of *Gossypium* species, accounting for only 17.39% (16/92). *Arabidopsis thaliana* constituted 1.09% (1/92) in this clade, while *Hypnum curvifolium* showed the highest proportion (23/92, 25.0%). Strikingly, Clade II exclusively contained *Gossypium*-specific ABI3 paralogs, suggesting clade-restricted divergence events within this clade. Quantitative analysis of ABI3 gene distribution across plant evolutionary grades revealed distinct expansion trends (Figure 1B). Algal species exhibited minimal ABI3 representation, with *Chlorokybus atmophyticus* encoding only three homologs. Bryophytes displayed remarkable intra-group variation: *Hypnum curvifolium* harbored 69 ABI3 genes, whereas *Marchantia polymorpha* retained merely 9. Notably, the Angiosperm *Sapindus mukorossi* (12 genes) showed comparable ABI3 numbers to bryophytes (*Anthoceros* spp.: 12–14 genes).

Meanwhile, Diploid model species (*Oryza sativa* and *Arabidopsis thaliana*) maintained equivalent ABI3 complements (14 vs. 13 genes), potentially reflecting conserved functional requirements in diploid systems. Within *Gossypium*, tetraploid species (e.g., Gh) retained approximately twofold higher ABI3 numbers than diploid progenitors (except Ghe), indicative of post-polyploidization retention dynamics. Gt possessed the most immense ABI3 repertoire among cotton species, while Ghe showed the most pronounced contraction.

### 3.2. ABI3 Chromosome Localization in Eight Cotton Species and Their Physico-Chemical Characteristics

**Figure 2:**
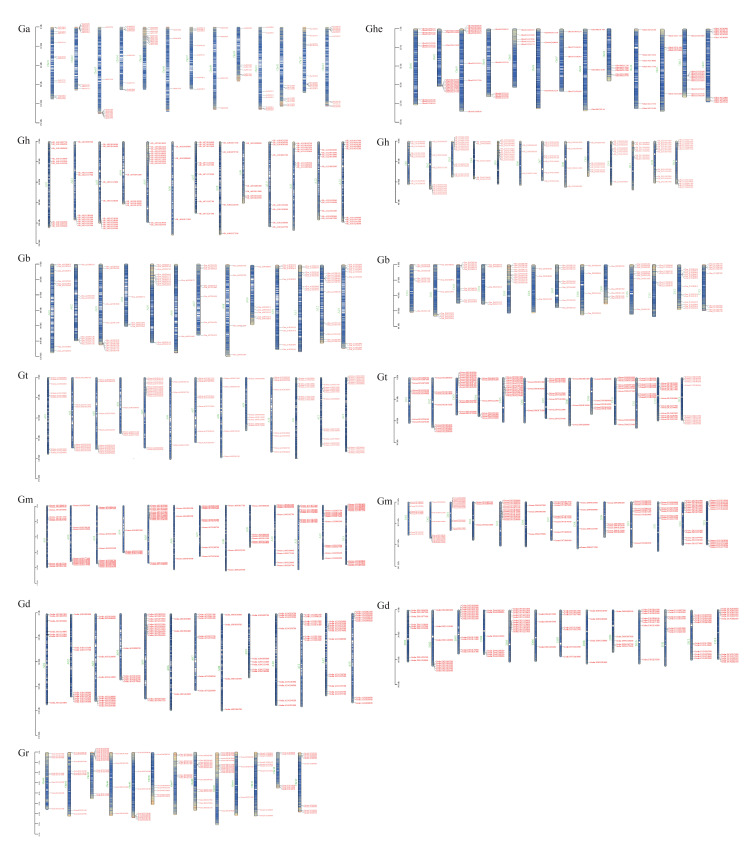
Location of the ABI3s gene on the chromosomes of eight cotton species.

Comparative analysis of ABI3 gene distribution across eight cotton species revealed conserved chromosomal localization patterns. Chromosomal mapping using Tbtools demonstrated predominant enrichment of ABI3 genes at chromosome termini (Figure 2). Minimal gene counts (n = 3) were observed on 16 chromosomes across species, including Ga_Chr06, Ghe_Chr06, and Gr_Chr10. Notably, Gr_Chr03 and Gt_D03 exhibited maximum gene density (n = 14). Distinct distribution patterns emerged between diploid and tetraploid species. Diploid genomes showed maximal inter-species variation on Chr08 (Ga/Ghe/Gr = 4/13/3) and minimal variation on Chr13 (Ga/Ghe/Gr = 10/11/10). Tetraploid genomes displayed relatively uniform chromosomal distributions, with the most significant divergence on D03 (nChr = 3 genes; Gt/Gh/Gb/Gm/Gd = 14/12/11/12/12). Physicochemical characterization revealed substantial molecular diversity (Supplementary Table S3): protein length varied 16.4-fold (93–1526 aa). Molecular mass ranged from 10,675.44 Da (Ghe02G20680) to 172,164.2 Da (Ga12G2364). Isoelectric points spanned 4.49 (Gbar_A09G010230) to 10.51 (Ghe02G20470). Instability indices indicated Gh_D02G238000 as most stable (14.93) versus Godar.A06G096200 as least stable (77.11). Hydrophobicity extremes ranged from −1.128 (Gbar_D13G003670) to 0.275 (Ghe10G32190). This comprehensive analysis establishes ABI3 as a structurally diverse gene family with conserved chromosomal localization patterns across cotton species, suggesting potential functional divergence through evolutionary adaptation.

### 3.3. Evolutionary Analysis of the ABI3 Gene Family Synteny in Cotton

**Figure 3:**
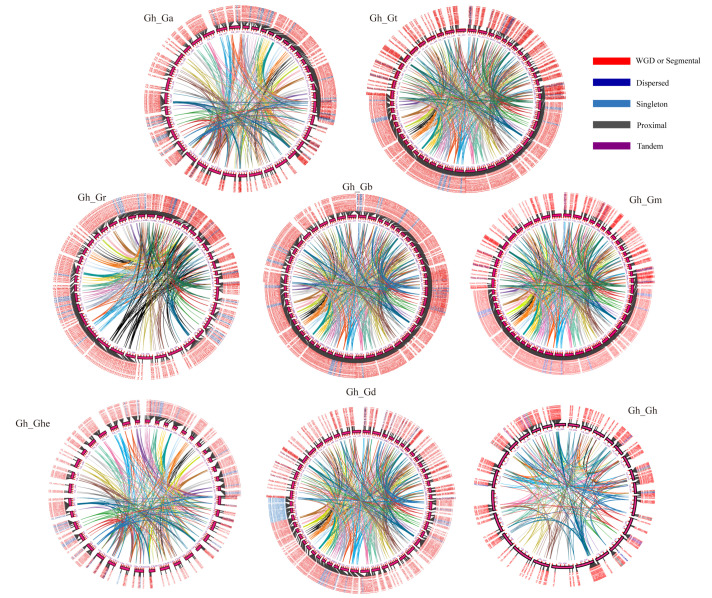
Analysis of ABI3s gene collinearity between land cotton and Ga, Ghe, Gr, Gb, Gt, Gm, and Gd cotton species. Different colors inside the circle indicate blocks of colinearity between Gh and genes of different cotton species. Different colors outside the circle indicate various ways of gene replication: red indicates whole-genome replication, dark blue indicates dispersed replication mode, blue color indicates singleton replication, gray indicates proximal replication, and purple indicates the tandem mode. Distinct colors within the circles indicate syntenic relationships between *G. hirsutum* ABI3 genes and their orthologs in other *Gossypium* species (with reference to the standardized color scheme in Supplementary Table S4).

Gene duplication drives functional diversification and adaptive evolution in plants [32]. To elucidate duplication patterns of the ABI3 family, we analyzed collinearity within Gh and between Gh and Ga, Ghe, Gb, Gr, Gt, Gm, and Gd using Tbtools’ MCScanX module (Figure 3). The results demonstrated that whole-genome duplication (WGD) was the predominant mode of expansion (89.71%) for the ABI3 family in Gh, followed by proximal duplication (4.41%) and tandem duplication (3.92%). Similar to the expansion pattern in Gh, whole-genome duplication (WGD) was the primary expansion mode for the ABI3 family across other cotton species. In contrast to Gh, however, secondary expansion modes included dispersed duplication (9.8%) and singleton duplication (3.4%). This WGD-dominated expansion aligns with established duplication mechanisms in cotton gene families, suggesting conserved evolutionary trajectories for ABI3 homologs [33,34].

### 3.4. Comparative Analysis of ABI3 Gene Family Evolution and Structural Features in Upland Cotton

**Figure 4:**
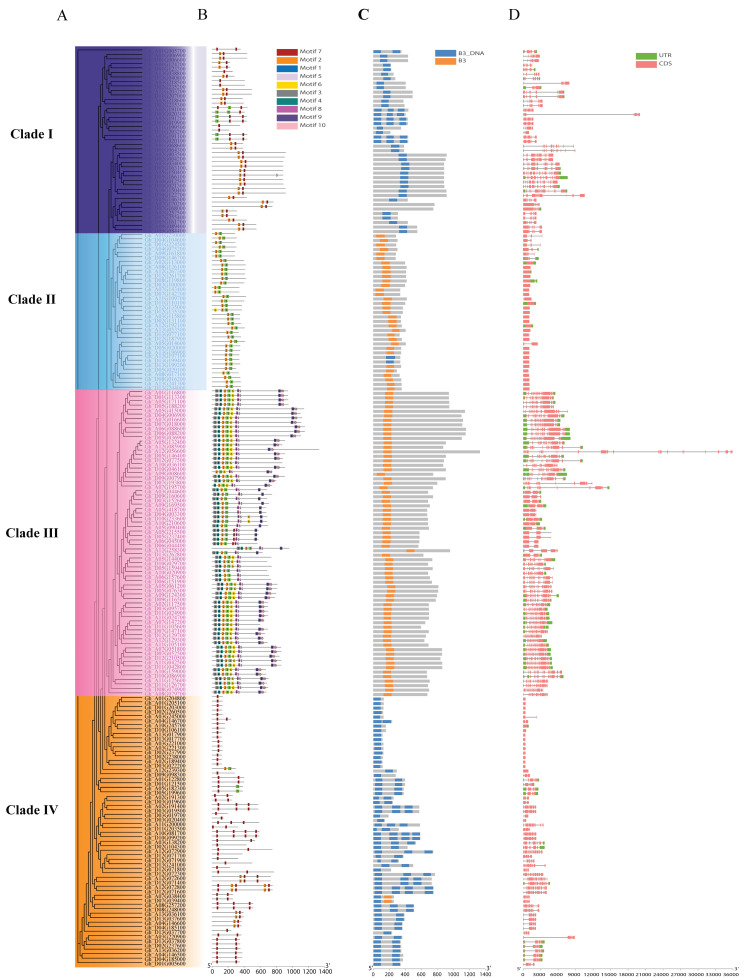
Phylogenetic analysis, motif analysis, conserved domain analysis, and gene structure analysis of the GhABI3s gene family in upland cotton. (**A**) Phylogenetic tree of 204 genes by IQ-TREE. (**B**) Motifs analysis. (**C**) Conserved domain analysis. (**D**) Gene structure of the GhABI3s gene family.

Phylogenetic and structural characterization of 204 GhABI3 genes revealed distinct evolutionary patterns and functional diversification. The genes clustered into four clades (Figure 4A), with Clade I containing the largest subgroup (n = 81) and Clade II the smallest (n = 20). MEME analysis identified 10 conserved motifs (Figure 4B and Figure S1), where motif 2 exhibited the highest conservation (detected in 141 genes), contrasting with motif 10 (47 genes). The motif distribution correlated strongly with phylogenetic clade: clade I: dominates motif 7, clade II: primarily dominates motif 2 and motif 7, clade III contains motif 1 and motif 2, while clade IV mainly includes motifs 1–6 and also motifs 8–10. It is surprising that the proportion of motif 7 is only 5.88% in clade IV. Domain analysis via CDD classified all GhABI3 genes into two categories: 50% containing the B3-DNA domain and 50% the B3 domain. Notably, *Gh_A07G038400* and *Gh_D07G039400* uniquely possessed both domains. Gene structure analysis revealed exon numbers ranging from 1 to 16 (excepting *Gh_A12G056600*); on average, each gene contains 6.9 exons. Among these, *Gh_A12G056600* possessed the highest number of exons and the longest sequence (26 exons spanning 35,945 bp). Approximately 19.11% of genes contained only a single exon, while genes with 10 or more exons accounted for 34.80% of the total (Supplement Table S5). The variation in exon numbers may be closely associated with functional divergence. Interestingly, the number of exons is closely linked to the quantity of motifs—the greater the number of exons, the more diverse the types of motifs they tend to contain. Comparative analyses of the evolutionary relationships of the proteins, conserved motifs, and gene structures indicate that the ABI3 gene may have multiple functions.

### 3.5. Promoter Cis-Element Profiling of GhABI3 Genes

**Figure 5:**
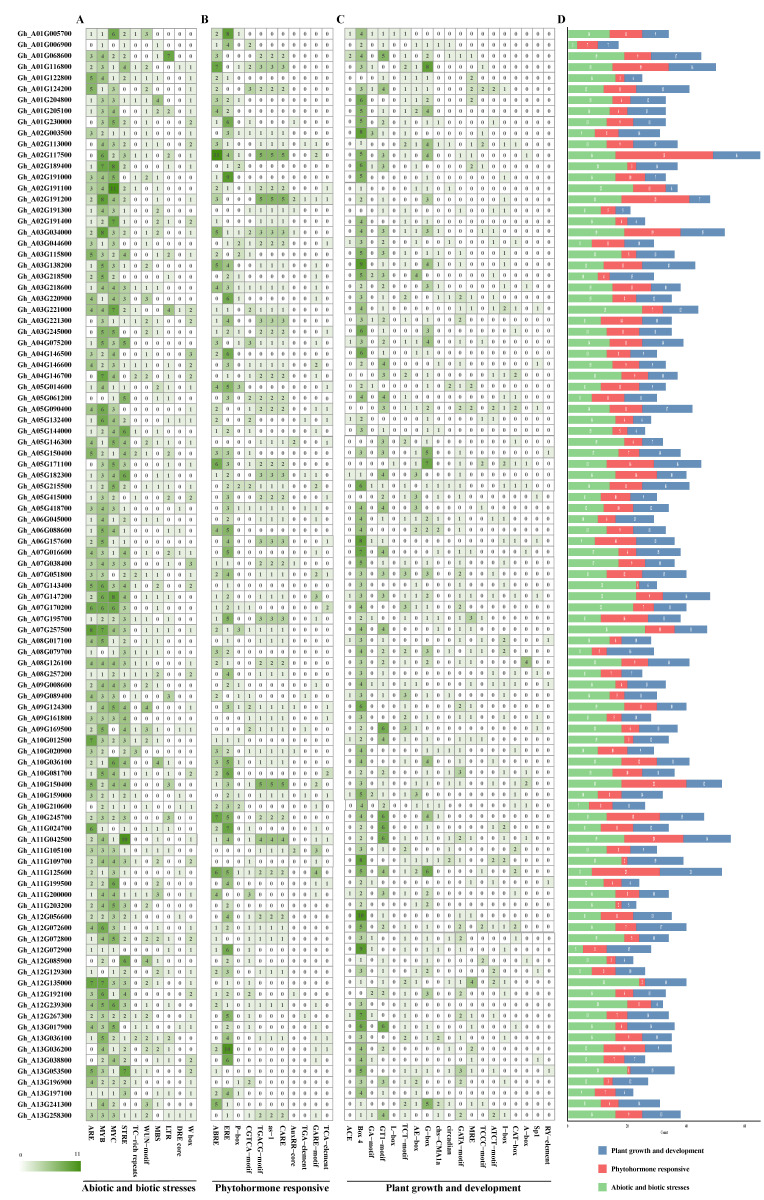
Analysis of cis-acting elements in the promoters of A_t_ subgroup genes in the GhABI3s gene family. (**A**) Abiotic and biotic stress elements. (**B**) Phytohormone-responsive elements. (**C**) Plant growth and development elements. (**D**) Proportion of different categories in genes.

**Figure 6:**
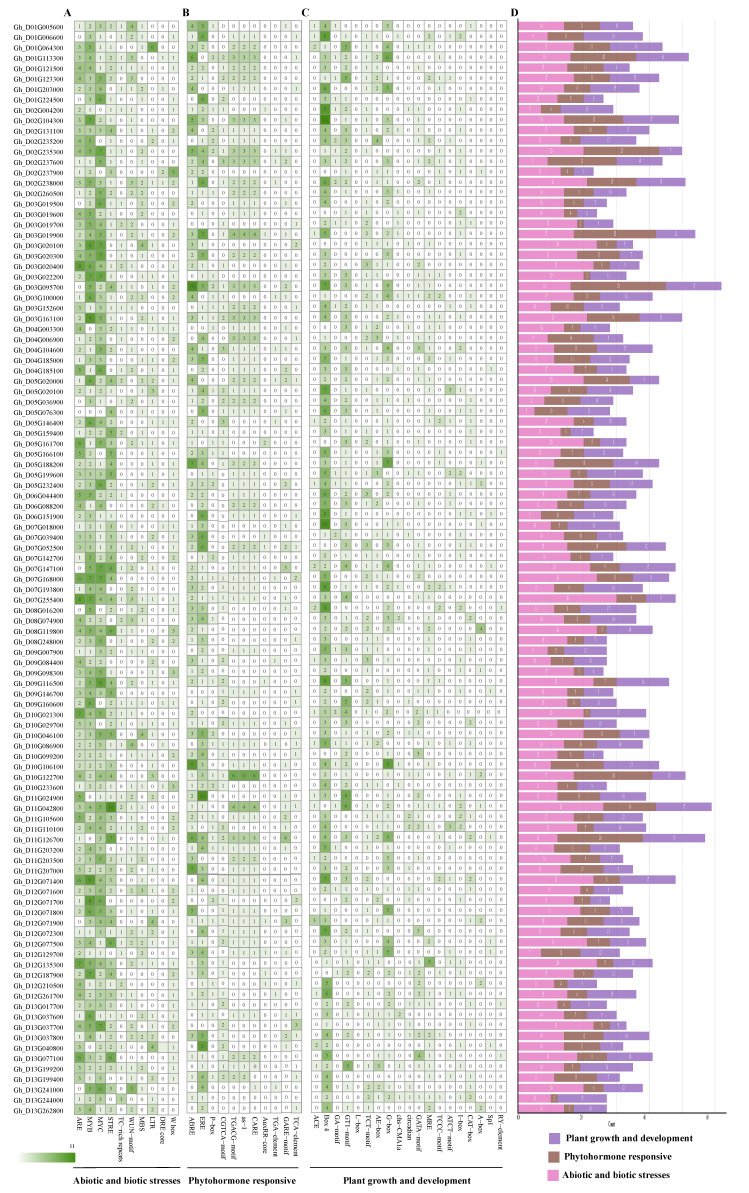
Analysis of cis-elements in the promoters of D_t_ subgroup genes in the GhABI3s gene family. (**A**) Abiotic and biotic stress elements. (**B**) Phytohormone-responsive elements. (**C**) Plant growth and development elements. (**D**) Proportion of different categories in genes.

The analysis of 2000 bp promoter regions upstream of GhABI3 genes using PlantCARE identified three functional cis-element categories: (1) stress-responsive (3043 elements), dominated by MYB-binding sites (49.75%, 1514/3043), with minimal DRE-core representation (0.89%, 27/3043); (2) hormone-responsive (1886 elements), featuring ethylene-responsive elements (ERE, 26.30%, 496/1886) and rare auxin-related motifs (AuxRR-core, 1.06%, 20/1886); and (3) growth/development-associated (2371 elements), enriched in Box 4 (30.11%, 714/2371) but depleted in RY-elements (0.38%, 9/2371) (Supplementary Table S6). At-sub-genome genes exhibited fewer elements across all categories than Dt-sub-genome homologs (Figure 5D and Figure 6D). Extreme divergences included *Gh_D03G019500* (65 stress elements; 33 hormone elements) versus *Gh_D01G224500* (3 stress elements) and *Gh_D12G071400* (21 growth elements) versus *Gh_D13G017700* (4 growth elements). Strikingly, fiber-specific *Gh_D08G016200* harbored only one hormone-responsive element, suggesting specialized regulatory minimalism. These cis-element landscapes highlight GhABI3’s adaptive regulatory diversity, with MYB-driven stress responses and sub-genome-biased element distribution potentially underpinning environmental adaptability in cotton.

### 3.6. Tissue-Specific Expression Profiling of GhABI3 Genes in Cotton Fiber Development

Transcriptomic analysis of GhABI3 genes in two cotton cultivars with contrasting fiber quality—J02-508 (high quality) and ZRI-015 (low quality)—revealed spatiotemporal expression patterns linked to developmental specialization. Ubiquitous expression across roots, stems, leaves, and fibers highlighted both conserved and divergent regulatory roles. Constitutive regulators *Gh_A12G192100* and *Gh_D12G187900* exhibited high expression in the roots and stems of both cultivars, suggesting core functions in vegetative growth, while *Gh_A10G245700* and *Gh_D10G106100* displayed broad tissue expression, implying pleiotropic roles. Fiber-specific activation of *Gh_D08G016200* (5–25 DPA) without expression in vegetative tissues underscored its exclusive contribution to fiber elongation. Organ-preferential expression was observed for *Gh_A11G042500* and *Gh_D11G0242800*, predominantly in leaves and ovules, potentially regulating reproductive processes. Notably, J02-508 showed elevated expression of leaf-associated *Gh_A12G192100* (3.2-fold higher than ZRI-015), suggesting cultivar-specific regulatory adaptations underlying fiber quality divergence. These findings establish GhABI3 as a multifunctional gene family coordinating developmental and fiber-specific programs, with *Gh_A10G245700* and *Gh_D10G106100* emerging as prime targets for fiber trait enhancement.

## 4. Discussion

The ABI3 gene family, conserved across angiosperms and gymnosperms, is essential for coordinating seed maturation, dormancy, early seedling establishment, the flowering process, and abiotic stress responses [3,35,36,37]. ABI3 proteins act as central regulators in ABA signaling, directly activating genes involved in lipid synthesis, desiccation tolerance, and stress adaptation [7,38]. Functional studies reveal their roles in balancing hormonal cross-talk (e.g., auxin, brassinosteroid) and epigenetic modifications to fine-tune developmental plasticity [6,37,39]. Notably, ABI3 homologs enhance drought and cold tolerance by modulating osmotic regulation and ROS scavenging [40,41].

The ABI3 family in plants plays a crucial role in growth, development, and response to biotic and abiotic stresses. The ABI3 gene family is poorly studied in plants. In the present study, 1927 ABI3 genes were identified and analyzed among 26 species. These genes were used to classify ABI3 into 10 distinct clades, with Clade I including the most significant number of species, while Clade II contained only eight cotton species, suggesting possible functional divergence (Figure 1A). Meanwhile, the number of genes of ABI3 in the same group of species remained consistent, suggesting that it is highly conserved in the same group of species. Still, there were also some species with differences in the number of genes of the same group of species, which may be caused by differences in their adaptations to the environment or the relatively limited number of species analyzed (Figure 1B); this is consistent with previous research [42]. The number of ABI3 genes in Arabidopsis is 86, in rice it is 80, and in tetraploid cotton, the number of ABI3 genes is 2.5 times that of Arabidopsis and cotton, which may be caused by polyploidization or whole-genome duplication.

Gene structure and motif analyses provide essential information about phylogenetic relationships and are closely related to protein function. Members of the GhABI3 gene family within the same subgroup have similar gene structures and motif compositions, suggesting they may have similar roles in plant growth and development. In contrast, the different motif types and quantitative differences between groups may be related to functional diversity. The substantial enrichment of motif 7, alongside the deletion of other motifs in Clade IV and the loss of motif types in Clades I and II, may reflect sub-genome-specific evolutionary pressures driving the adaptive expansion of upland cotton [43,44]. Gene structure not only governs functional properties but also contributes critically to the regulation of gene expression. In eukaryotic organisms, the distribution frequency, length, and nucleotide composition of exons and introns exhibit substantial interspecies divergence [45]. In humans, each gene comprises an average of eight exons, whereas the average length of introns is approximately 5850 base pairs (bp) [46]. Conversely, research on Arabidopsis thaliana and rice (*Oryza sativa*) indicates that the average number of exons per gene is six and five, respectively. Furthermore, the average size of introns in Arabidopsis is about 170 bp, compared to an average of 447 bp for introns in rice. This variation in intron size and exon number across species may reflect differing evolutionary pressures and adaptations to their respective environments, highlighting the diversity of gene architecture in flowering plants [47,48]. The average number of exons in the cotton ABI3 gene family is approximately 6.9. Notably, *Gh_A12G056600* exhibits the highest number of exons, totaling 26. Additionally, 19.12% of the genes within this family consist of a single exon. Previous studies have shown that the phenomenon of intron retention can lead to the premature termination of gene transcripts, resulting in the inability of normal transcripts to be translated into complete proteins. This process may subsequently contribute to the manifestation of different phenotypes [49]. The above results suggest that genes with more exons and introns may be more likely to encode proteins that are functionally complex, finely regulated, and require diversity through mechanisms such as alternative splicing. Despite a comprehensive characterization of the ABI3 family, our study shares limitations common to annotation-dependent genomics. Structural characterization of the ABI3 gene family and cis-element identification in promoters are inherently constrained by the completeness and accuracy of reference genome annotations. Potential inaccuracies may arise from assembly gaps, fragmented gene models, or static annotation files that cannot capture dynamic regulatory contexts [50]. To mitigate these limitations, future studies should integrate long-read transcriptomics (e.g., PacBio Iso-Seq) for full-length gene model validation, complemented by chromatin accessibility profiling (ATAC-seq) and functional verification via CRISPR-Cas9-mediated cis-element mutagenesis [51,52,53,54]. Such multi-omics approaches would bridge computational predictions with in vivo biological validation.

**Figure 7:**
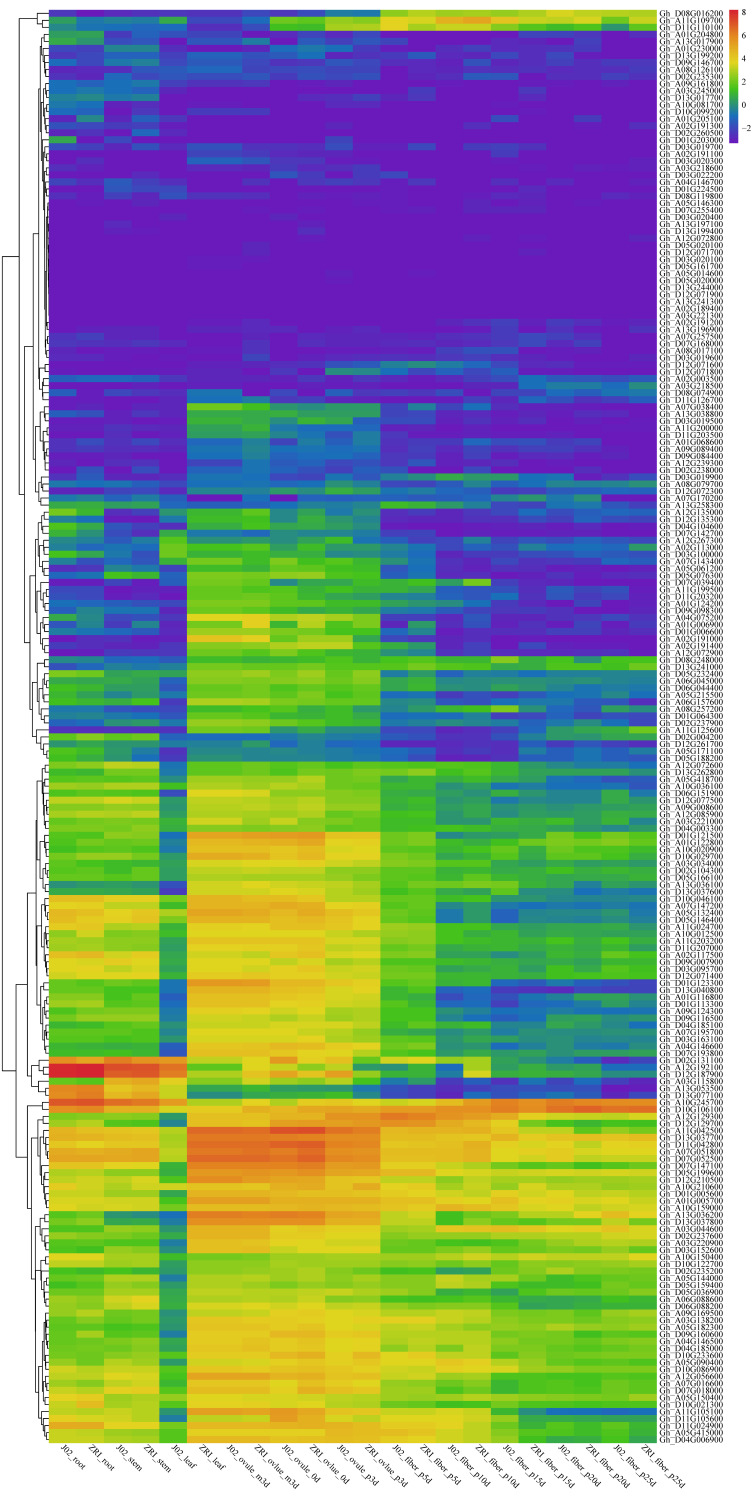
Heatmap of GhABI3 gene expression in different groups of J02-508 and ZRI-015. Differentially expressed genes of Gh_ABI3s genes under root, stem, leaf, ovules, and fiber.

Previous studies have shown that co-expression of ABI3/ABI5 in cotton significantly enhances the drought resistance of cotton ABI3 [40]. The environmental adaptability of upland cotton, a key factor contributing to its global dominance as a fiber crop, may be partially attributed to ABI3-mediated developmental plasticity. Tissue-specific expression profiling revealed similar expression patterns of GhABI3 homologs (e.g., *Gh_A12G192100* and *Gh_D12G187900* in roots; *Gh_A10G245700* in stem; and *Gh_D10G106100* in developing fibers) (Figure 7). While previous studies established that abscisic acid (ABA) negatively regulates fiber elongation, our observation of upregulated expression of *GhD10G106100* in both cultivars presents an apparent contradiction. This discrepancy is likely attributable to methodological differences between experimental approaches. As a transcription factor, ABI3 modulates downstream gene expression and consequently regulates seed dormancy and vernalization through direct binding to cis-regulatory elements within target gene promoters. Therefore, bioinformatic prediction of cis-regulatory elements in gene promoter regions provides a valuable strategy for inferring potential biological functions.

Tissue-specific expression patterns are strongly associated with the composition of promoter regulatory elements. Analysis of the promoters of *Gh_D03G019500* and *Gh_D12G187900* revealed a predominance of putative stress-responsive elements, with counts of 65 and 17 elements associated with biotic and abiotic stress, respectively. We thus propose that *Gh_D03G019500* and *Gh_D12G187900* function as transcriptional regulators of *G. hirsutum*’s stress-response pathways under variable environments. Elements related to growth and development constituted the second most abundant category (n = 11 and n = 12, respectively). In contrast, the promoter of *Gh_D10G106100* was primarily enriched for elements implicated in growth, development, and hormonal responses. This differential enrichment of regulatory elements aligns with the established role of multiple hormones in regulating fiber development [55]. The above results also suggest that the ABI3 gene may be involved in both vegetative growth and fiber development, with the latter serving as a model system for studying cell elongation and expansion. Notably, homoeologous GhABI3 gene pairs exhibited coordinated expression dynamics across developmental stages and tissues, implying potential functional compensation between sub-genomes (Figure 7).

## 5. Conclusions

In this study, we analyzed the phylogenetic analysis of 1927 ABI3 protein sequences spanning 26 species that revealed ubiquitous conservation of ABI3 across diverse taxa. Further investigation of the ABI3 gene family in cotton (*Gossypium* spp.) identified lineage-specific expansion, primarily driven by whole-genome duplications (WGD). A comprehensive characterization of ABI3 genes in upland cotton—encompassing gene structure, conserved domains, promoter *cis*-elements, and spatiotemporal expression patterns—suggests that structural features and promoter architecture likely underpin tissue-specific regulation. Collectively, these findings demonstrate that ABI3 genes are highly conserved yet exhibit lineage-specific diversification in cotton, where they play pivotal roles in environmental adaptation and fiber development.

## Data Availability

The original contributions presented in this study are included in the article and Supplementary Materials. Further inquiries can be directed to the corresponding author(s).

## Supplementary Materials

The following supporting information can be downloaded at https://www.mdpi.com/article/10.3390/genes16080854/s1: Figure S1: Sequence information of 10 conserved motifs in ABI3 proteins of *G. hirsutum*; Table S1. Accession codes and download links for all 27 species’ genomic resources; Table S2. ABI3 gene family clades information for 27 species; Table S3. Comparative biochemical characterization of ABI3 transcription factors across eight *Gossypium* Species; Table S4. Chromosome identifier-color correspondence in *G. hirsutum*; Table S5. Exon number of the ABI3 gene in *G. hirsutum*; Table S6. Frequency distribution of stress-responsive cis-acting elements in the ABI3 promoter (*G. hirsutum*).
