# Supplementary figures and images for: Genome-Wide Characterization of the ABI3 Gene Family in Cotton
Source: Genes (Basel). 2025 Jul 23;16(8):854. doi: 10.3390/genes16080854 (PMC12385726; doi:10.3390/genes16080854)

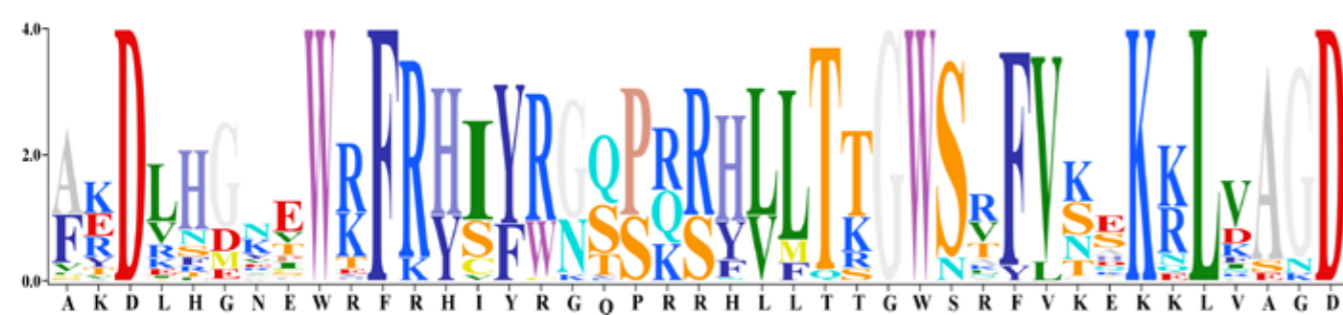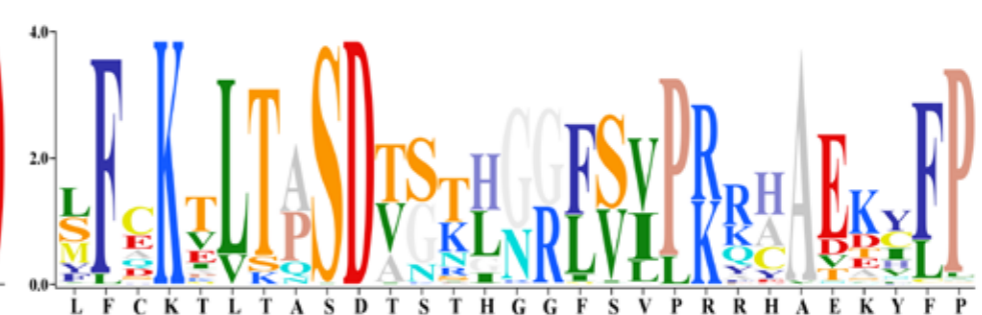

|          |         |
|----------|---------|
| Motif 1  | Motif 2 |
| Motif 3  | Motif 4 |
| Motif 6  | Motif 5 |
| Motif 9  | Motif 7 |
| Motif 10 | Motif 8 |

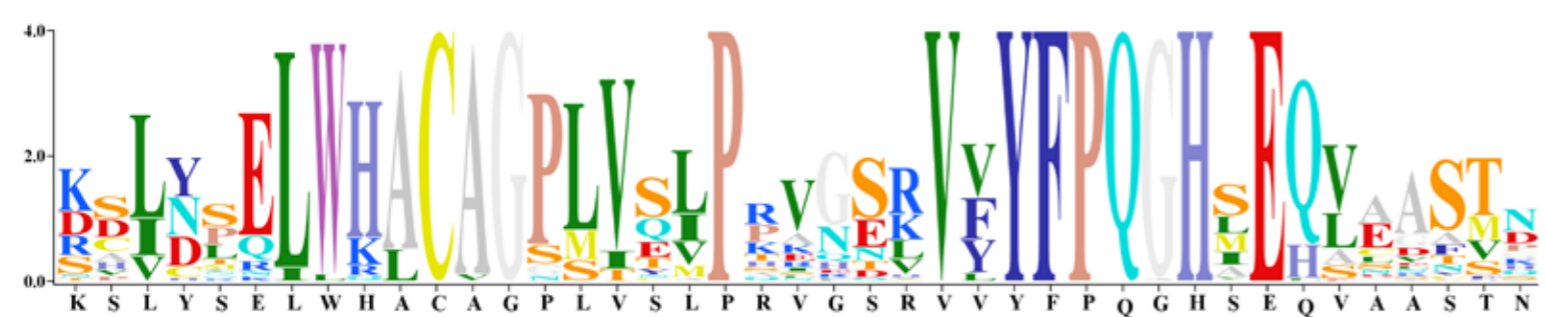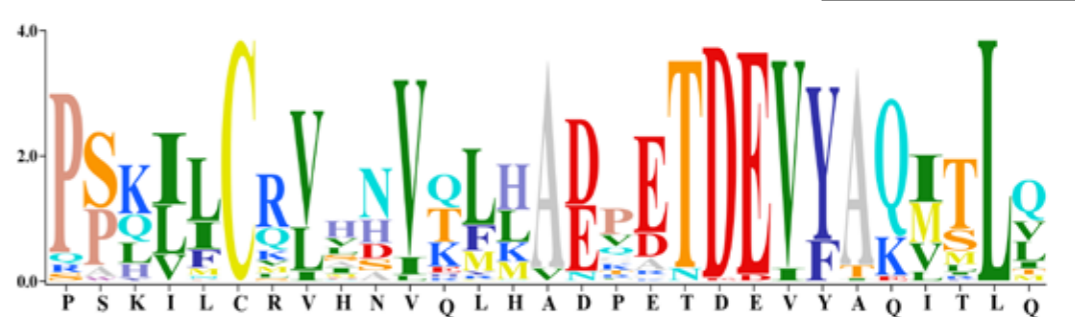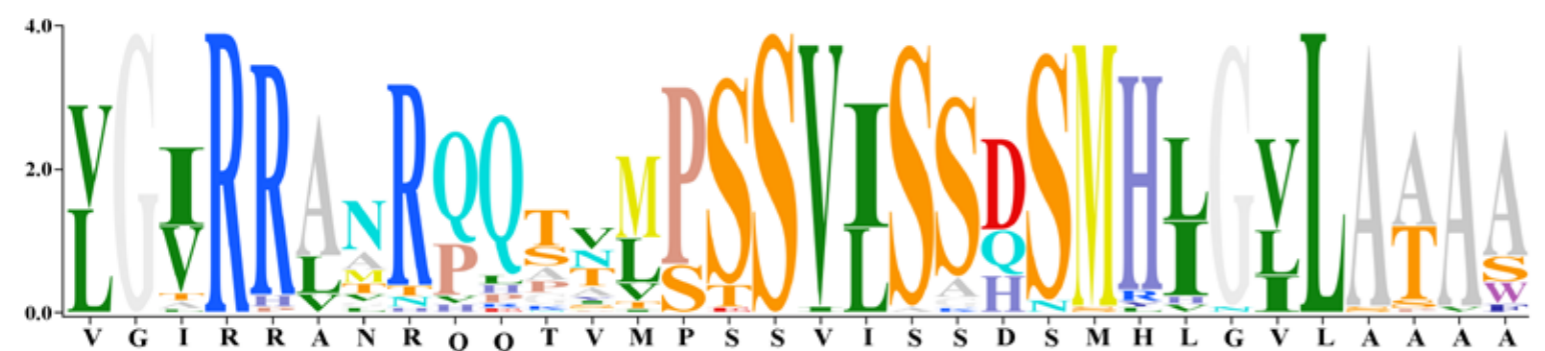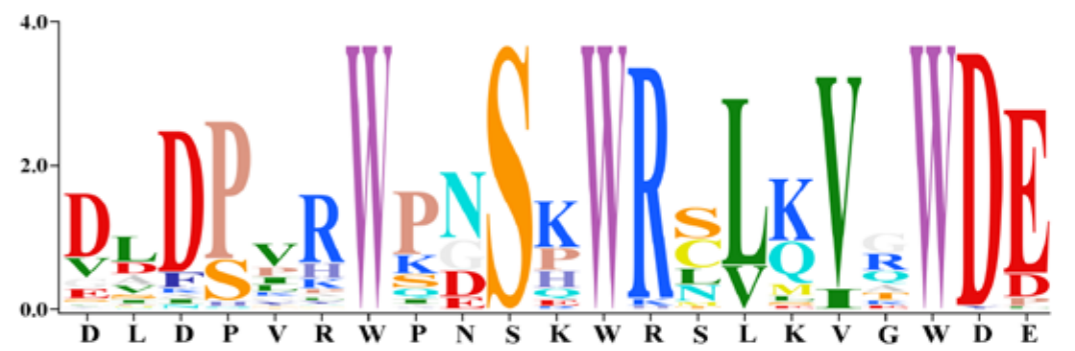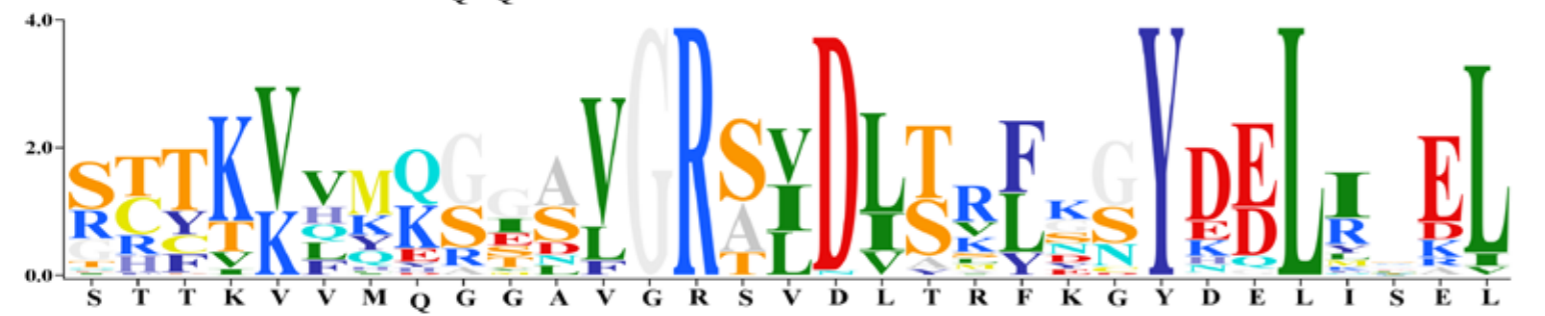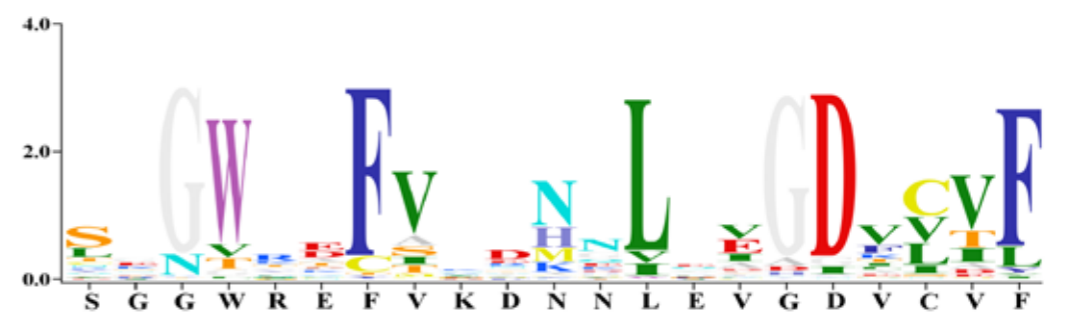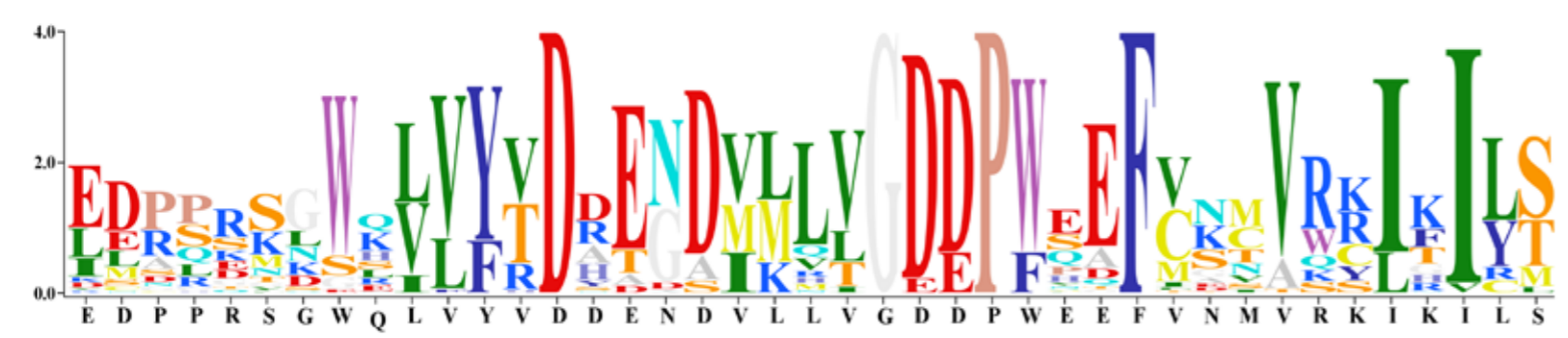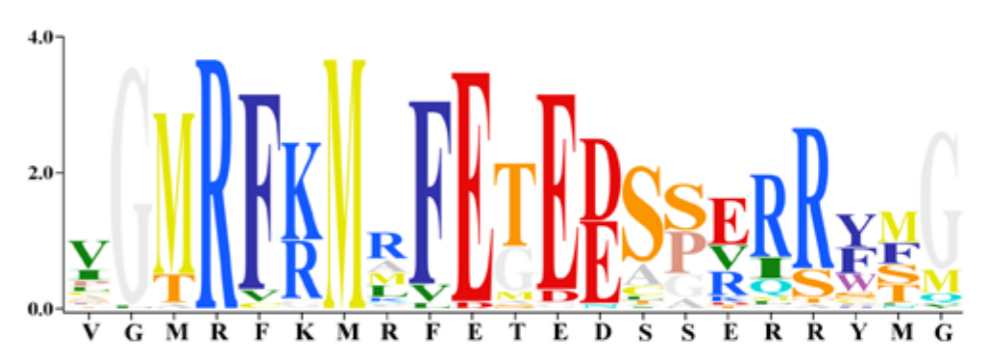

Supplement: Supplementary file 1 [file genes-16-00854-s001.zip › genes-3742686-supplementary/Figure S1.pdf]
